# Physical activity and risk of workplace and commuting injuries: a cohort study

**DOI:** 10.5271/sjweh.4163

**Published:** 2024-09-01

**Authors:** Maria Alhainen, Mikko Härmä, Jaana Pentti, Jenni Ervasti, Mika Kivimäki, Jussi Vahtera, Sari Stenholm

**Affiliations:** 1 Unit of Public Health, University of Turku and Turku University Hospital, Turku, Finland.; 2 Centre for Population Health Research, University of Turku and Turku University Hospital, Turku, Finland.; 3 Work Ability and Work Careers, The Finnish Institute of Occupational Health, Helsinki, Finland.; 4 Clinicum, Faculty of Medicine, University of Helsinki, Helsinki, Finland.; 5 Department of Mental Health of Older People, Faculty of Brain Sciences, University College London, London UK.; 6 Research Services, Turku University Hospital and University of Turku, Turku, Finland.

**Keywords:** injury prevention, occupational health, occupational injury, prospective cohort study, public sector employee

## Abstract

**Objectives:**

Leisure-time physical activity (PA) has been hypothesized to reduce the likelihood of occupational injuries, but it is unclear whether this association varies between workplace and commuting injuries. The aim of this study was to examine the association between PA and risk of workplace and commuting injuries.

**Methods:**

Data were derived from the Finnish Public Sector study including 82 716 person-observations (48 116 participants). PA was requested repeatedly in four questionnaire surveys between 2000–2012. The average level of PA from two subsequent questionnaires was used to assess long-term PA. To obtain a 1-year incidence of injuries, participants were linked to occupational injury records from the national register. Logistic regression analysis with generalized estimating equations was used to examine the association between PA and injury risk. The analysis was adjusted for age, sex, education, work schedule, job demand, sleep difficulties, cardiovascular diseases, diabetes, and depression for workplace and commuting injuries, and workplace injuries were additionally adjusted for physical heaviness of an occupation and injury risk by occupation.

**Results:**

Higher level of PA was associated with a lower risk of workplace injuries compared to inactive participants [odds ratio (OR) 0.85, 95% confidence interval (CI) 0.73–0.98]. This association was most marked in the ≥50-year-old age group (OR 0.78, 95% CI 0.64–0.99). No association between the PA and the risk for commuting injuries was observed.

**Conclusions:**

Higher PA is associated with lower risk of workplace injuries particularly among older employees.

Occupational injuries can have long-term health consequences affecting the ability to work and perform daily activities during leisure time (1, 2). Moreover, the costs of occupational injuries for the society are high (3). In order to prevent occupational injuries, tackling modifiable factors that affect the risk of occupational injury is important.

Observational studies have identified several risk factors for occupational injuries, including older age, female gender, lower education, physical and psychosocial work stressors, work schedule and chronic health problems, such as sleep difficulties, diabetes, cardiovascular diseases, and depression (4–15). The mechanisms underlying these conditions vary by risk factor. For example, in manual jobs, in which the exposure to physical hazards is greater, some workers may lack the physical capacity to meet the physical requirements of the work, especially older workers with declining physical functioning (5, 16). Even though men have more workplace injuries than women due to different occupations and tasks at work, women may be at a higher risk for work-related injury and musculoskeletal diseases after taking into account occupational factors such as job title and physical demands of the occupation. In general, due to physiological differences between the sexes, the average female worker is exposed relatively to greater physical demands than the average male worker in job tasks requiring a high level of force exertion (6).

Because physical activity (PA) has many beneficial effects on health and functioning, it may also have the potential to reduce the risk of injuries. In general, PA and exercising to improve muscle strength, balance and cardiorespiratory fitness as well as weight-bearing activities help to maintain bone density (17, 18). These health benefits are typically achieved after long periods of moderate-to-vigorous levels of PA and may help prevent falls, fractures, and other injuries (19, 20). In addition, engaging regularly in moderate-to-vigorous level PA is associated with reduced risk for diseases such as type 2 diabetes and depression, helps with weight control, and improves sleep, all of which are known to increase the risk of occupational injuries (21–23).

A recent systematic review on this topic identified 11 studies examining the relationship between physical fitness and occupational injuries among emergency responders. The authors concluded that good cardiovascular fitness may protect from occupational injuries (24). However, a majority of the reviewed study populations were young men with a mean age of 30–40 years. In addition, the jobs of emergency responders (police, firefighters, ambulance workers) are physically demanding, and include several external hazards, which increase the risk for injuries. Thus, further studies with wider age groups and varying occupations are warranted to clarify the role of PA on injury risk and identify subgroups who are at increased risk of occupational injuries.

To date, relatively few studies have directly examined the association between PA and commuting injuries. One previous study examined the association between active commuting and commuting injury risk and found that commuting by bicycle was associated with a higher risk of transport-related injuries and admission to hospital compared to non-active modes of commuting (25). However, the study focused on how different means of commuting modes are associated with the risk of injuries; and, as commuting might be a large proportion of employees' daily PA, more information on the association between the overall PA level with commuting injury risk is needed.

This study aimed to examine the association between long-term PA with workplace and commuting injuries in a wide range of occupations among public sector workers in general and by age and sex. Commuting injuries were included because commuting on foot or by bicycle can be an important component of daily PA for many employees, but also a cause of commuting accidents.

## Methods

### Study population

The data were derived from the Finnish Public Sector study (FPS), which is a prospective cohort study of public sector employees (26). The participants responded to questionnaires on work and lifestyle, including PA in survey waves conducted in 2000–2002 (N=48 598, response rate 68%), 2004 (N=48 076, response rate 66%), 2008 (N=52 891, response rate 71%) or 2012 (N=53 133, response rate 69%). Participants, who answered the questions on PA in two consecutive surveys (2000 and 2004; 2004 and 2008; 2008 and 2012) were included in the study; and, in total, the analytic sample consisted of 82 716 person observations and 48 116 participants. Those who lacked information of PA in two consecutive surveys, were excluded from the study.

Participants were linked to injury records obtained from the Federation of Accident Insurance Institution from 1 January 2000 until 31 December 2015.

The Ethics Committee of the Hospital District of Helsinki and Uusimaa approved the FPS (HUS 1210/2016).

### Assessment of physical activity

Participants were requested to estimate their average weekly hours of leisure-time and commuting PA during the previous year in activities corresponding to walking, brisk walking, jogging, and running. Each intensity level had five response options of which the class mid-points were used for the calculation of time spent in PA per week: no activity, <0.5 hours (15 minutes used for the calculation), ~1 hour (45 minutes), 2–3 hours (2.5 hours), and ≥ 4 hours (5 hours). Time spent at each intensity level was multiplied by the average energy expenditure of each activity, expressed in metabolic equivalent of tasks (MET) (27, 28). Each intensity level, walking, brisk walking, jogging, and running, corresponds to 3.5, 5, 8, and 11 MET hours, respectively. The total amount of PA was quantified as weekly MET hours per week by summing up the amount of activity at each intensity level together.

To capture long-term PA, the average level of PA of two subsequent surveys (four-year interval, 2000 and 2004; 2004 and 2008; 2008 and 2012) was computed. This approach also allows multiple inclusions of each participant if they responded to at least two consecutive surveys.

The participants were categorized into four groups based on the average level of reported leisure-time and commuting PA of two subsequent surveys: 'inactive' (<7 MET hours/week), 'low active' (7–14 MET hours/week), 'moderately active' (14–30 MET hours/week) and 'high active' (> 30 MET hours/week) (29, 30).

### Assessment of workplace and commuting injuries

Occupational injury was defined as an injury to the employee caused by an accident attributable to an unexpected, sudden event at the workplace or during commuting by any means (for example by foot, bicycle, means of public transportation, or car). The injuries (for example wounds and superficial injuries, dislocations, sprains, strains, bone fractures) are reported to the employer. According to the Finnish legislation, the employer is required to take out an insurance for all employees in case of occupational injuries and occupational diseases and all injuries and diseases are compensated through a statutory insurance system. Reported workplace or commuting injuries are recorded in the national register maintained by the Federation of Accident Insurance Institutions and are collected into the statistics (31). The national personal identification numbers (unique code assigned to all Finnish residents) were used to link the cohort members of the FPS study to these records until 31 December 2013. For the current study, we used a 1-year-follow-up after each study wave to capture relatively recent injuries after the measurement of PA.

### Covariates

The covariates used in the analyses have been shown to be associated with occupational injuries (4, 6–9, 11, 12, 15, 32). Age and sex were derived from employers' records. Age was classified into two categories: <50 and ≥50 years.

The employee's highest degree of education was derived from data from the Central Statistical Office Finland and classified into three categories: primary, secondary, or tertiary.

Work schedule was self-reported and was categorized into 'regular working time' (only day shifts) and 'shift work' (shift work with or without night shifts).

To assess the physical heaviness of different occupations, a gender-specific job exposure matrix (JEM) designed to be used in epidemiological studies was used to assess physical exposures (33). The physical heaviness of an employee's occupation was categorized into 'high' or 'low' based on the JEM.

To assess the risk of injury by occupation, a new variable was created, which shows the number of workplace injuries in different occupations per thousand working years during the year 2000. The specific number of injuries in different occupations per thousand employee years is shown in table 1.

**Table 1:** The injury risk by occupation per thousand working years.

Occupation	Number of injuries/1000 working years during year 2000
Doctor	4
Senior advisor in municipal administration	4
Senior advisor in other position	5
Department secretary	7
Techical specialist	8
Psychologist, therapisy	8
Librarian	9
Social worker	9
Office worker	10
Director of education	12
Financial secretary, communications officer	12
Specialist in other position	12
Lecturer, teacher	13
Director in other position	15
Public health nurse and other nurses	15
Head nurse	17
Day care teacher	19
Artist, reporter	19
Physiotherpist occupationa therapist	21
Director of health care and social services	22
Service industry	23
Class teacher	23
Family day carer	24
Dental hygienist	24
Day care worker	25
Special education teacher	25
Social advisor, youth worker	26
Nurse, practical nurse	28
Technical specialist	32
Other employee	32
Home care assistant	38
Kitchen supervisor, cook	40
Orderly	41
Operator	48
Kitchen assistant	56
Park worker	68
Assisstant	73
Classroom assistant	74
Real estate manager	80
Buildin worker, repair worker	86
Firefighter, security guard	107

Job demands were measured using the shorter version of the Job Content Questionnaire and was based on three statements: "I have to work really hard", "I am expected to perform excessive amount of work" and "I do not have time to get my job done". Each participant's mean response (scale 1–5) was calculated (34, 35).

The information on participants' sleep difficulties was obtained from questionnaires, where the frequency (never, one night per month, one night per week, 2–4 times per week, 5–6 nights per week, and nearly every night) of each type of sleep difficulty ('waking up too early, 'having trouble staying asleep', 'feeling fatigued or drowsiness despite a typical night's rest' and 'difficulty to fall asleep') was classified according to Jenkins Sleep Problem Scale (36). Sleep difficulties were categorized into two groups based on the most frequent symptom: 'no sleep difficulties' (≤4 nights per week) or 'sleep difficulties' (≥5 nights per week). The categorization captures those who exceed the diagnostic criteria for an insomnia disorder (37).

In Finland, medicines necessary for the treatment of certain serious and long-term diseases can have special reimbursements. In these cases, the Social Insurance Institution covers the costs of the medicine. In this study, the identification of diabetes was based on the special reimbursement of diabetes drugs (oral or insulin) and the identification of cardiovascular diseases was based on the special reimbursements of coronary heart disease drugs or if the participant had been subject to hospital care due to coronary heart disease (11). The information on a hospital care episode was obtained from the Finnish Institute of Health and Welfare's national register on hospitalizations maintained. If the participant had been in hospital care with diagnoses code I6 or I20-I21 (myocardial infarction or coronary heart disease), the participant was considered to have been admitted to hospital due to the coronary heart disease.

Information about whether the participant had depression was based on the survey question: "Has your doctor ever told you that you have depression?" (yes or no).

### Statistical analyses

Characteristics of the study population are presented for the whole study population and for each PA group separately. We described categorical variables as numbers and percentages and continuous variables as means and standard deviations (SD). The differences in characteristics in different PA groups were tested using a chi-squared test for categorical variables and the analysis of variance was used to calculate means and SD for continuous variables.

The association between different PA groups and workplace and commuting injuries, which occurred within the 1-year period after the assessment of long-term PA, was tested using logistic regression analyses with generalized estimating equations (GEE). The GEE model controls the intra-individual correlation between the repeated measurements. The standard errors were computed using the robust ('sandwich') estimators, which is the default for SAS 9.4.

To examine the role of age and sex on the association between PA groups and occupational injuries, age and sex interactions with PA were tested in the logistic regression analyses with GEE, and the results were shown by age groups and sex. As older employees and women are more prone to occupational injuries than younger employees and men due to physiological differences, we wanted to examine does PA decrease the risk of occupational injuries especially in these risk groups (4, 38).

Results are shown as odds ratios (OR) and 95% confidence intervals (95% CI). The analyses were initially adjusted for age (continuous), sex and education (Model 1) when examining both workplace and commuting injuries. The second model (Model 2) was additionally adjusted for work-related factors (work schedule, physical heaviness of an occupation, injury risk by occupation and job demands when examining workplace injuries, and work schedule and job demand when examining commuting injuries, as physical heaviness of an occupation and injury risk by occupation do not affect directly the commuting injury risk) and the last model (Model 3) was additionally adjusted for health-related factors (sleep difficulties, cardiovascular diseases, diabetes, depression for both workplace and commuting injuries).

All the analyses were conducted by using SAS statistical software, version 9.4, SAS Institute, Cary, NC, USA.

## Results

The characteristics of the study population and different PA levels are shown in table 2. The mean number of measurements of long-term PA was 1.72 (range 1–3 depending on the number of subsequent waves available). The mean age of the study population was 49.2 (SD 8.3) years. There were differences by age groups, so that moderate or high PA were more common among participants aged <50 years, whereas inactivity and low PA were more common among participants aged ≥50 years. Among women, the greatest proportion were moderately active whereas among men, inactivity or high level of PA were more common.

**Table 2:** Characteristics of the study population in different physical activity groups. [MET=metabolic equivalent of tasks; SD=standard deviation.]

		All			Level of physical activity											P-value
					Inactive (<7 MET hrs/wk)			Low (7–14 MET hrs/wk)			Moderate (>14–30 MET hrs/wk)			High (>30 MET hrs/wk)		
		N (%)	Mean (SD)		N (%)	Mean (SD)		N (%)	Mean (SD)		N (%)	Mean (SD)		N (%)	Mean (SD)	
		82 716 (100)			8582 (10)			15 371 (19)			30 397 (37)			28 366 (34)		
Age																
	<50 years	38 886 (47)			3366 (39)			6314 (41)			13 926 (46)			15 280 (54)		<0.0001
	≥50 years	43 830 (53)			5216 (61)			9057 (59)			16 471 (54)			13 086 (46)		
Sex																
	Men	15 223 (18)			1855 (22)			2613 (17)			4831 (16)			5924 (21)		<0.0001
	Women	67 493 (82)			6727 (78)			12 758 (83)			25 566 (84)			22 442 (79)		
Education																
	Primary	6147 (7)			1062 (12)			1367 (9)			2037 (7)			1681 (6)		<0.0001
	Secondary	28 665 (35)			3477 (41)			5577 (36)			10 296 (34)			9315 (33)		
	Tertiary	47 904 (58)			4043 (47)			8427 (55)			18 064 (59)			17 370 (61)		
Physical heaviness of an occupation																
	Low	70 443 (85)			7024 (82)			13 030 (85)			26 135 (87)			24 254 (86)		<0.0001
	High	11 614 (15)			1494 (18)			2222 (15)			4009 (13)			3889 (14)		
	Missing	659			64			119			253			223		
Work schedule																
	Regular working time	58 476 (71)			6129 (72)			11 101 (73)			21 795 (72)			19 451 (69)		<0.0001
	Shift work	23 414 (29)			2358 (28)			4092 (27)			8303 (28)			8661 (31)		
	Missing	826			95			178			299			254		
Sleep difficulties																
	Yes	21 047 (25)			2746 (32)			4220 (28)			7663 (25)			6418 (23)		<0.0001
	No	61 525 (75)			5813 (62)			11 119 (72)			22 686 (75)			21 907 (77)		
	Missing	144			23			32			48			41		
Cardiovascular diseases																
	Yes	1083 (1)			200 (2)			262 (1)			370 (1)			251 (23)		
	No	81 633 (99)			8382 (98)			15 109 (99)			30 027 (99)			28 115 (77)		<0.0001
Diabetes																
	Yes	1983 (2)			421 (5)			549 (4)			638 (2)			375 (1)		<0.0001
	No	80 733 (98)			8161 (95)			14 822 (96)			29 759 (98)			27 991 (99)		
Depression																
	Yes	10 284 (13)			1454 (18)			2247 (16)			3773 (13)			2810 (10)		<0.0001
	No	68 182 (87)			6530 (82)			12 237 (84)			25 107 (87)			24 308 (90)		
	Missing	4250			598			887			1517			1 254		
Injury risk by occupation/1000 working years			23.3 (18.1)			26.0 (19.9)			23.5 (18.0)			22.6 (1.7)			23.2 (18.6)	<0.0001
Job demands (1–5)			3.22 (0.89)			3-23 (0.92)			3.24 (0.92)			3.23 (0.88)			3.21 (0.88)	<0.0001

In all, 2477 workplace injuries and 1304 commuting injuries were recorded during the 1-year follow-up period. Of the commuting injuries, 793 (61%) occurred while travelling to work by foot, 366 (28%) by bicycle, 109 (8%) by car, moped or motorbike, and 36 (3%) by other commuting methods (eg, bus, train, tram, boat or other commuting method).

Table 3 shows that the risk for workplace injury was lower in the moderate (OR 0.88, 95% CI 0.79–1.00) and the high active (OR 0.84, 95% CI 0.74–0.96) groups compared to the inactive group after adjusting for age, sex, and education. After taking into account work-related factors (work schedule, physical heaviness of an occupation, injury risk by occupation and job demands), the association remained essentially the same for both groups (OR 0.88, 95% CI 0.77–1.00 and OR 0.82, 95% CI 0.71–0.94). Further adjustment for health-related factors (sleep difficulties, cardiovascular diseases, diabetes, depression) diluted the association for moderately active compared to the inactive, but the association remained significant in the high active group (OR 0.85, 95% CI 0.73–0.98).

**Table 3:** The association between physical activity (PA) and workplace injuries during 1-year follow-up. [N=total observations in the group; n=workplace injuries in the group; OR=odds ratio; CI=confidence interval.]

	Incidence			Model 1 ^a^			Model 2 ^a^			Model 3	
	N/n	%		OR	95% CI		OR	95% CI		OR	95% CI
Inactive	8582/314	3.7		1 (ref)			1 (ref)			1 (ref)	
Low PA	15 371/489	3.2		0.93	0.81–1.08		0.93	0.80–1.07		0.95	0.81–1.11
Moderate PA	30 397/875	2,9		0.88	0.79–1.00		0.88	0.77–1.00		0.91	0.79–1.04
High PA	28 366/802	2.8		0.84	0.74–0.96		0.82	0.71–0.94		0.85	0.73–0.98

^a^ Model 1 is adjusted for age, sex and education. ^b^ Model 2 is adjusted for age, sex, education, work schedule, physical heaviness of an occupation, injury risk by occupation and job demand. ^c^ Model 3 is adjusted for age, sex, education, work schedule, physical heaviness of an occupation, injury risk by occupation, job demand, sleep difficulties, cardiovascular diseases, diabetes and depression.

**Figure 1:**
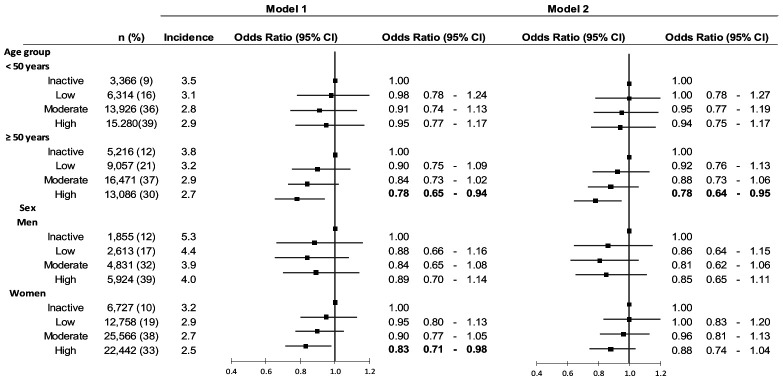
The association between physical activity (PA) and workplace injuries across age and sex groups. Model 1 is adjusted for age, sex, and education. Model 2 is adjusted for age, sex, education, work schedule, physical heaviness of an occupation, injury risk by occupation, job demand, sleep difficulties, cardiovascular diseases, diabetes and depression.

The association between PA and workplace injuries by age and sex are shown in figure 1. In general, the incidence of workplace injuries among men (4%) was higher compared to women (3%), whereas there was no difference in the overall injury incidence between those <50 (3%) and those ≥50 (3%) years. The ≥50-year-old and women in the high active groups had a significantly lower risk for workplace injury compared to the inactive group in Model 1 (OR 0.78, 95% CI 0.65–0.94 and OR 0.83, 95% CI 0.71–0.98). After adjusting for work- and health-related factors (Model 2), the results remained significant among ≥50-year-old (OR 0.78, 95% CI 0.64–0.95). However, no sex or age group interaction with PA on workplace injuries was observed.

**Figure 2:**
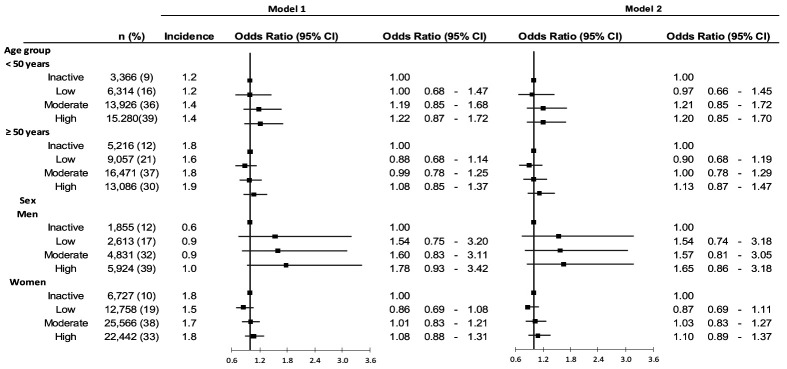
The association between physical activity (PA) and commuting injuries across age and sex groups. Model 1 is adjusted for age, sex, and education. Model 2 is adjusted for age, sex, education, work schedule, physical heaviness of an occupation, injury risk by occupation, job demand, sleep difficulties, cardiovascular diseases, diabetes and depression.

Table 4 shows the association between PA and commuting injuries. No statistically significant associations were observed between the level of PA and commuting injury risk. In the age and sex specific subgroup analysis, no statistically significant associations between PA and commuting injury risk were found (figure 2).

**Table 4:** The association between physical activity (PA) and commuting injuries during 1-year follow-up. [N=total observations in the group; n=commuting injuries in the group, CI=confidence interval.]

	Incidence			Model 1 ^a^			Model 2 ^b^			Model 3 ^c^	
	N/n	%		OR	95% CI		OR	95% CI		OR	95% CI
Inactive	8582/135	1.6		1 (ref)			1 (ref)			1 (ref)	
Low PA	15 371/220	1.4		0.92	0.74–1.14		0.91	0.74–1.13		0.93	0.74–1.16
Moderate PA	30 397/489	1.6		1.06	0.88–1.29		1.05	0.87–1.28		1.07	0.88–1.32
High PA	28 366/60	1.6		1.14	0.94–1.38		1.13	0.93–1.38		1.15	0.94–1.41

^a^ Adjusted for age, sex and education. ^b^ Adjusted for age, sex, education, work schedule and job demand. ^c^ Adjusted for age, sex, education, work schedule, job demand, sleep difficulties, cardiovascular diseases, diabetes and depression.

## Discussion

In this prospective study of 48 116 public sector employees and 82 716 observations, higher long-term level of PA was associated with a lower risk for workplace injuries compared to inactive employees. The association was particularly pronounced in employees aged 50 years. No association was observed between the level of PA and commuting injuries.

To our best knowledge, this was the first large prospective cohort study of a wide variety of occupations that examined the association between PA and workplace and commuting injuries. Unlike other studies which measured participants' physical fitness, we focused on PA during commuting and leisure time. Our results are in line with previous findings from smaller scale studies, which have suggested that good physical fitness may protect from injuries in physically demanding jobs, such as emergency responders and especially among older workers (4, 24, 39). Although self-reported leisure-time PA serves only a proxy for physical fitness, our findings extend previous evidence by suggesting that long-term high level of PA may decrease the risk for workplace injuries also in occupations which are not physically demanding.

Our subgroup analysis found that ≥50-year-old highly active workers had a significantly lower risk for workplace injuries compared to inactive workers. PA might decrease the risk of an injury via multiple mechanisms, including, for instance, improving or maintaining balance, increasing aerobic fitness, and enhancing or maintaining muscle strength. This is particularly important among older workers as physical functioning declines with advancing age, but regular higher level PA helps to maintain these features (16). However, regular and versatile PA already during childhood and adolescence years may also contribute to long-term musculoskeletal health and injury resilience in adulthood. Thus the role of PA in injury prevention may be important across the entire lifespan (40, 41).

In addition to better physical fitness among highly active workers, other health-related factors could explain the association between a higher level of PA and a lower workplace injury risk. Firstly, those who are more active in the long term are more often normal weight and have fewer chronic diseases, factors that are also shown to be associated with lower injury risk. In addition, a clear dose–response relationship has been reported between higher level of PA and longer chronic disease-free life expectancy (11, 22, 45, 46). Secondly, as sleep difficulties and depression are known risk factors for occupational injuries, the injury risk could be decreased by PA's beneficial effect on sleep and mental health (21, 23, 47, 48). However, in the current study, the associations between PA and workplace injuries remained significant after adjusting for health-related factors suggesting that the high level of PA lowers the injury risk also independently.

Contrary to previous studies, we observed no association between the level of PA and commuting injury risk (25). Previous studies have suggested that employees who are physically highly active may be more likely to commute by foot or bicycle, which predisposes them to accidents and injuries more than those who travel to work by public transportation or car. However, a possible explanation for dissimilar results in our study is that we were not able to separate leisure-time and commuting PA because participants were asked to report total PA during leisure time, including commuting. To disentangle the role of leisure-time and commuting PA on commuting injuries, further studies with detailed information about types and contexts of PA are needed.

This study has several strengths. We had reliable information on workplace and commuting injuries from the national register, which is connected to the statutory insurance system and includes all employer-reported injuries. Additionally, the study population consisted of public sector employees from various occupations and not only employees in, for example, physically demanding jobs. We were able to consider several work-related factors, which may influence the injury risk in various occupations, such as the physical heaviness of different occupations and the average injury rate per occupation as well as work schedule and psychosocial job demands. PA was assessed from two time-points to obtain an estimate of long-term PA.

The present study had also several limitations. First, the level of PA was self-reported, and participants may have over- or underestimated their accurate time engaged in PA. Participants were asked to assess their time engaged in each level of PA during leisure time and commuting corresponding to walking, brisk walking, jogging, and running. There are also several other types of PA than walking or running in which participants can engage, such as bicycling or skiing. It may have been difficult for the participants to assess their real time engaged in different levels of PA thus the level of PA could have been underestimated. However, self-assessed PA and MET using similar short instruments, such as accelerometers, have been widely used in previous epidemiological studies to achieve more accurate measurement, but these would be difficult and expensive to use in large populations (49). Moreover, we measured PA based on repeated measurements, which we have shown better captured cardiometabolic risks in this cohort than a single measurement of the most recent activity level (30). Second, public sector jobs are relatively safe and working conditions and safety are strictly regulated in Finland. This may limit the generalizability of the results. Third, the occupational injury occurred during a 1-year-period after the assessment of PA, and thus we do not know how active the employee was at the time the injury occurred. However, often the beneficial effects of PA on health occur in the long term, and we tried to assess the average level of employees' PA through repeated measurements (30).

Our results suggest that the role of regular PA in workplace injury risk prevention should be emphasized at the workplace among employees and employers. Different health and exercising programs at workplaces tailored to aging workers may be useful in reducing and preventing workplace injuries, but effectiveness of such interventions warrants intervention studies.

In conclusion, higher PA was associated with lower risk of workplace injuries, especially among older employees. There was no significant association between the level of PA and commuting injuries, but further studies are needed to confirm the absence of association. Further studies are needed to examine specific mechanisms leading to increased injury risk among physically inactive workers as well as to study the effectiveness of PA interventions on workplace injury prevention.

## Data Availability

We are allowed to share anonymized questionnaire data of the FPS study with bona fide researchers with an established scientific record and bona fide organizations upon application. For information about the FPS study contact Dr. Jenni Ervasti (jenni.ervasti@ttl.fi).
